# Isolated muscular tuberculosis: unusual location of the Koch bacillus

**DOI:** 10.11604/pamj.2017.26.158.11795

**Published:** 2017-03-16

**Authors:** Zeineb Alaya, Walid Osman

**Affiliations:** 1 Department of Rheumatology, Farhat Hached Hospital, Faculty of Medicine of Sousse, Sousse, Tunisia; 2 Department of Orthopaedics, Sahloul Hospital, Faculty of Medicine of Sousse, Sousse, Tunisia

**Keywords:** tuberculosis, quadriceps muscle, MRI, biopsy, antituberculosis drugs

## Image in medicine

A 23-year-old female patient with a family history of pulmonary tuberculosis and tuberculous spondylodiscitis was hospitalized for swelling of the left anterior-external surface of the left thigh, (A) with altered general status. Biology has shown a biological inflammatory syndrome. The ultrasound showed a collection opposite the great trochanter fusing towards the lodge of the gluteus muscle and forward towards the terminal part of the common chief ischio-psoas. MRI of the hips revealed an intra-muscular collection of the antero-lateral side of the thigh root, taking contrast in the periphery, involving the quadriceps muscle and tensor of facia lata, (B and C). The biopsy of the collection under ultrasound guidance brought back 30 cc of casein. The diagnosis of tuberculosis of the quadriceps muscle and tensor of the facia lata was based on the positivity of the tuberculin skin test, (D), the positivity of the PCR for Mycobacterium tuberculosis in the puncture fluid and histology showing multiple epithelioid granulomas centered by caseous necrosis. There were no other tuberculous sites. The patient was treated with antituberculosis drugs for 12 months with good progress. Muscular tuberculosis (MT) is a rare localization of the disease (0.01 to 2%). Its occurrence without associated bone involvement is exceptional. MT often simulates a muscular tumor. MRI contributes to the improvement of the sensitivity of the diagnosis of MT because it allows to specify the extent of the muscular lesions and to orient the site of the muscular biopsy. The diagnosis is essentially based on histology.

**Figure 1:**
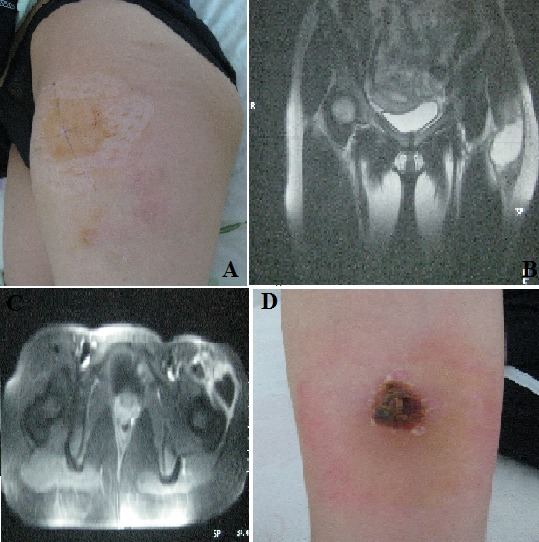
(A) swelling of the left anterior-external surface of the left thigh; (B, C) MRI of the hips: intra-muscular collection of the antero-lateral side of the thigh root, taking contrast in the periphery, involving the quadriceps muscle and tensor of facia lata; (D) tuberculin skin test positive with a phlyctenular appearance

